# Physiological phenotyping of dementias using emotional sounds

**DOI:** 10.1016/j.dadm.2015.02.003

**Published:** 2015-04-22

**Authors:** Phillip D. Fletcher, Jennifer M. Nicholas, Timothy J. Shakespeare, Laura E. Downey, Hannah L. Golden, Jennifer L. Agustus, Camilla N. Clark, Catherine J. Mummery, Jonathan M. Schott, Sebastian J. Crutch, Jason D. Warren

**Affiliations:** a Dementia Research Centre, UCL Institute of Neurology, University College London, London, UK; b London School of Hygiene and Tropical Medicine, University of London, London, UK

**Keywords:** Frontotemporal dementia, Alzheimer's disease, Emotion, Sound, Autonomic, Pupillometry

## Abstract

**Introduction:**

Emotional behavioral disturbances are hallmarks of many dementias but their pathophysiology is poorly understood. Here we addressed this issue using the paradigm of emotionally salient sounds.

**Methods:**

Pupil responses and affective valence ratings for nonverbal sounds of varying emotional salience were assessed in patients with behavioral variant frontotemporal dementia (bvFTD) (n = 14), semantic dementia (SD) (n = 10), progressive nonfluent aphasia (PNFA) (n = 12), and AD (n = 10) versus healthy age-matched individuals (n = 26).

**Results:**

Referenced to healthy individuals, overall autonomic reactivity to sound was normal in Alzheimer's disease (AD) but reduced in other syndromes. Patients with bvFTD, SD, and AD showed altered coupling between pupillary and affective behavioral responses to emotionally salient sounds.

**Discussion:**

Emotional sounds are a useful model system for analyzing how dementias affect the processing of salient environmental signals, with implications for defining pathophysiological mechanisms and novel biomarker development.

## 1 Introduction

Dementias are generally defined by progressive deterioration in cognitive function but often produce less well-characterized alterations in emotional, motivational, and social functions. These alterations are particularly early and significant in behavioral variant frontotemporal dementia (bvFTD) and semantic dementia (SD) within the frontotemporal lobar degeneration (FTLD) spectrum [1], [2], [3], [4], [5], [6], [7], [8], and are probably underrecognized in progressive nonfluent-aphasia (PNFA) [9] and Alzheimer's disease (AD) [4], [10], [11], [12]. However, although emotional disturbances are hallmarks of many dementias and potentially relevant to disease detection, tracking and therapy, the pathophysiology of disturbed emotion in dementia is poorly understood and challenging to measure objectively.

Particularly pertinent to the organization of emotional behaviors is the capacity to identify significant or “salient” objects and events in the external environment and to analyze the consequences of these for the individual's own homeostatic milieu. Emotionally salient stimuli may be linked to basic biological drives and are broadly relevant to social signaling, self-awareness, and reward seeking in a number of dementia syndromes [7], [13], [14], [15], [16], [17]. Autonomic responses index perceptual, cognitive, and emotional salience of sensory signals and normally require integrated neural network activity [18]. The large-scale brain networks targeted by neurodegenerative proteinopathies [19], [20], [21] traverse brain structures previously implicated in emotional salience processing: these include prefrontal and cingulate cortices, insula, mesial temporal and striatolimbic structures that evaluate significant internal and external sensory events, and effector regulatory mechanisms in basal forebrain and dorsal brainstem [8], [22], [23]. Taken together, this evidence suggests that the detailed characterization of altered autonomic and behavioral responses to emotionally salient stimuli might provide a basis for understanding and measuring the complex behavioral effects of dementia diseases [8].

The domain of nonverbal sounds includes highly salient biological signals that produce autonomic and other physiological effects. Altered processing of nonverbal sounds has been documented in a range of dementia diseases [2], [6], [24], [25], [26], [27], [28], [29], [30]. However, little information is available concerning the physiological correlates of processing nonverbal sounds (or indeed, other sensory stimuli) as salient sensory signals. Although autonomic dysfunction has been described in dementias [31], [32], few studies have assessed this systematically in relation to sensory salience coding. Patients with bvFTD and SD have been shown to have either normal or depressed autonomic reactivity to loud tones [13], [33] and more complex auditory and multimodal stimuli [34], [35] while autonomic reactivity may be retained in AD [33].

Here we took nonverbal sound as a model system to investigate systematically the physiological and behavioral correlates of processing sensory emotional salience in patients with canonical dementia syndromes. We used pupillometry to index autonomic (sympathetic) reactivity: compared with other candidate autonomic indices [36], [37], [38], pupil dilatation responses are relatively resistant to disease-associated movement and other artifacts, well preserved to auditory stimuli in healthy older individuals [39], track neural responses closely [23], [40], [41], and have social behavioral resonance [42], [43]. We used affective valence ratings to index the behavioral processing of auditory emotional salience. Three linked experimental hypotheses were tested: first, that dementia syndromes show profiles of altered physiological and affective responses to nonverbal sounds; second, that these syndromic profiles reveal dissociations between autonomic and affective behavioral indices of auditory emotional salience; and finally (and more specifically), these salience signatures stratify dementia syndromes associated with more severe clinical derangements of emotional processing (represented by bvFTD and SD) from clinically associated syndromes with the relative preservation of emotional responses (represented by PNFA and AD).

## 2 Methods

### 2.1 Participants

Forty-six patients fulfilling current consensus diagnostic criteria for dementia syndromes (14 bvFTD, 12 PNFA, 10 SD, 10 typical amnestic AD [5], [44], [45]; and 26 healthy age-matched individuals with no history of neurological or psychiatric illness participated. No participant had a clinical history of hearing loss or pupillary disease or clinical evidence of a mood disorder at the time of participation; to assess any effect from peripheral hearing function on experimental performance, screening pure tone audiometry was conducted in each group using a previously described procedure [46]. Ten patients with bvFTD had a genetic diagnosis (five pathogenic C9orf72 mutations, five MAPT mutations). Cerebrospinal fluid tau and beta-amyloid assays (available for a further 23 patients: six AD, seven bvFTD, four SD, six PNFA) and Florbetapir PET brain imaging (available for nine patients: six SD, three PNFA) further corroborated the clinical diagnoses (CSF total tau: beta-amyloid ratio >1 in all six AD cases and two PNFA cases, ratio <1 in other cases; Florbetapir-PET negative for amyloid deposition in available SD and PNFA cases). At the time of their participation, 18 patients were receiving treatment with acetyl-cholinesterase inhibitors (nine AD, six bvFTD, one SD, two PNFA), 12 with antidepressants (four bvFTD, three SD, three PNFA, two AD), and 2 with neuroleptic agents (both bvFTD).

All participants had a comprehensive assessment of general neuropsychological functions and patients had volumetric brain MRI in support of their syndromic diagnosis. In addition, nonverbal auditory semantic function was assessed in all participants using a novel semantic classification (matching) task on paired sounds that did not require verbal or other cross-modal labeling (see online Supplementary Material). General demographic and neuropsychological data for participant groups are summarized in Table 1. The experimental groups were well matched for age; males were significantly overrepresented in the byFTD group. Mean symptom duration was longer in the bvFTD group than other patient groups, reflecting the wide variation in disease tempo of patients with bvFTD; the syndromic groups were otherwise similar in overall disease stage. Average Mini-Mental State Examination (MMSE) was lower in the SD and AD groups than the healthy control group, but did not differ between patient groups.

All participants gave informed consent in accord with the principles laid down in the Declaration of Helsinki.

### 2.2 Experimental stimuli and procedures

#### 2.2.1 Sound stimuli

Based on affective valence and identifiability ratings obtained in a pilot experiment on a set of 180 common nonverbal sounds presented to healthy young adults, a subset of highly identifiable (environmental, animal, human, and mechanical) sound stimuli were selected, representing three emotional valence categories: “unpleasant” (e.g., a person spitting, a mosquito), “neutral” (e.g., telephone, throat clearing), and “pleasant” (e.g., baby laughing, stream burbling). Sound valence categories had similar overall identifiability ratings and sounds in each valence category were matched for other psychoacoustic properties. Final stimulus characteristics are described in Supplementary Tables 1 and 2 with further details in the online Supplementary Material. During the experiments, all sound stimuli were presented via high-fidelity headphones (ATH-M50 Audio-Technica^®^) from a notebook computer at a constant, comfortable listening level (at least 70 dB) in a quiet room.

#### 2.2.2 Pupillometry

**Fig. 1:**
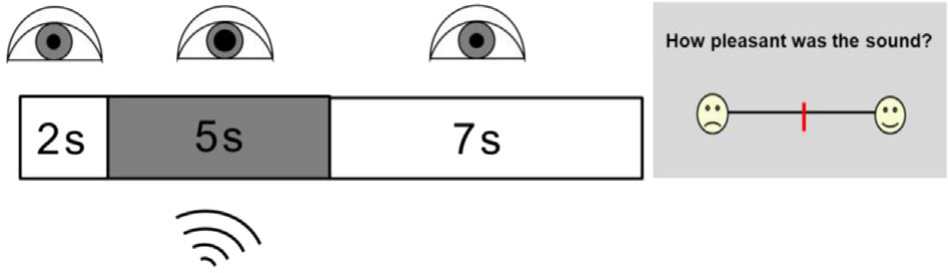
Schematic of trial design in the pupillometry experiment. Area of the right pupil was measured using a headset-mounted infrared camera, while the participant fixated the center of a monitor screen. Once stable fixation was achieved, a trial was triggered with an initial brief silent interval (2 seconds), followed by the sound stimulus (5 seconds; dark rectangle) and a final silent equilibration interval (7 seconds). On completion of the recording period, a Likert scale (right) was displayed and the participant was asked to rate the pleasantness of the sound on the line using a wireless mouse cursor; a response triggered the next recording period.

Pupil dilatation responses were measured for 27 sounds (nine from each valence category), presented in randomized order (see Supplementary Table 1); three additional sounds were presented as an initial familiarization set but not further analyzed. Trial design and pupil recording methodology are schematized in Fig. 1. On the completion of pupil recording for each trial, a modified Likert scale (Fig. 1) was displayed and the participant was asked to rate the pleasantness (affective valence) of the sound. All pupil response and behavioral rating data were stored for off-line analysis.

### 2.3 Data analysis

Pupillometric data were preprocessed (see online Supplementary Material) and all further data analyses were implemented using STATA12^®^. For all analyses, a threshold *P* < .05 was taken as the criterion for statistical significance.

Pupil response and behavioral affective valence rating data were compared between participant groups and group associations between pupil responses and valence ratings were assessed using linear regression models. The log ratio of maximal pupil area to baseline pupil area (pupil_max_) was derived as the metric of pupil response. Statistical models incorporated measured sound peak volume (as a surrogate for perceived loudness; online Supplementary Material) and gender as nuisance covariates. Variability within each group of individual pupil_max_ responses and affective valence ratings was assessed by calculating the difference between an individual's rating or pupil_max_ response and the mean for that group; linear regression models were used to compare participant groups.

For each participant group, we assessed associations between group mean pupil responses (pupil_max_) and group mean affective valence ratings using a regression model with mean pupil_max_ as the dependent variable and mean sound valence and (mean sound valence)^2^ as predictors, to capture any linear or quadratic association with pupil response (because pupil response was anticipated to increase both for highly positively and negatively valenced sounds). The statistical design took individual variation in pupil responses into account (details in online Supplementary Material). Measures of correlation strength (r^2^ values) between pupil response and affective valence were generated for each group.

Clinical symptom duration, MMSE score, and reverse spatial span (a cognitive measure of nonverbal executive function and working memory) were taken as surrogates of disease severity across syndromes and correlations of these disease measures, peripheral hearing function, and medication use with pupil reactivity and auditory affective valence ratings were assessed in the patient cohort. Relations between auditory affective ratings and performance on the nonverbal auditory semantic test were separately assessed (online Supplementary Material).

## 3 Results

### 3.1 Behavioral affective valence rating profiles

Mean affective valence ratings assigned to each sound by the healthy older control group and the healthy young pilot control group were strongly positively correlated (r^2^ = 0.96, *P* < .0001; valence ratings assigned to the sound stimuli by all groups are listed in Supplementary Table 1). Healthy older individuals did not differ significantly in the variance of their valence ratings over the sound set (see Supplementary Fig. 1).

**Fig. 2:**
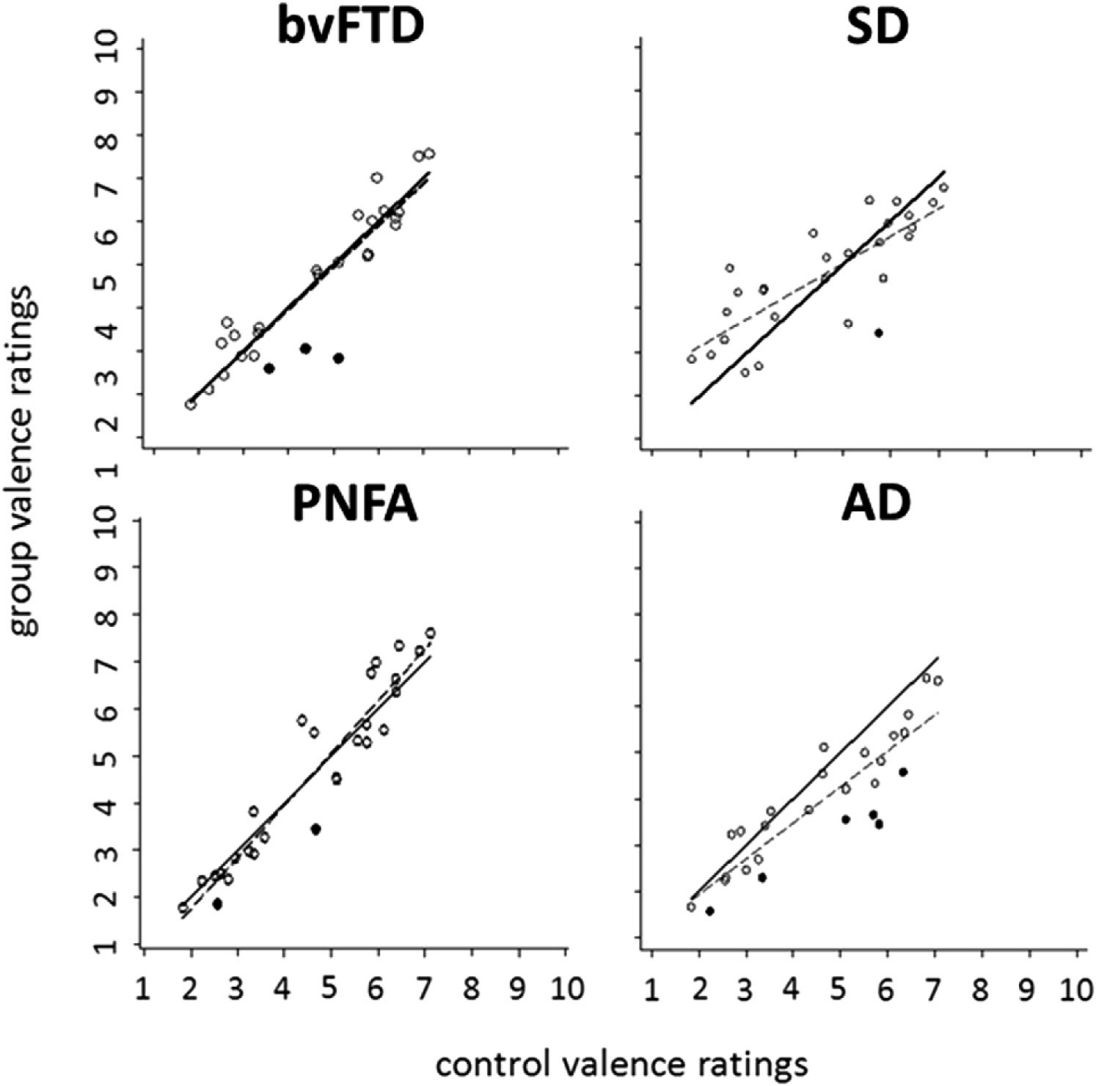
Mean group affective valence (pleasantness) rating for each stimulus sound plotted against healthy older control group mean affective valence ratings, for each patient group. Ratings are on a Likert scale where 1 and 10 indicate most unpleasant and most pleasant, respectively. For ease of visualization, lines of best fit for control group ratings (solid line) and patient group ratings (dashed line) are plotted. Black filled squares code particular sounds for which mean valence ratings were significantly different (*P* < .05) between patients and healthy older controls. AD, Alzheimer's disease; bvFTD, behavioral variant frontotemporal dementia; PNFA, progressive nonfluent aphasia; SD, semantic dementia.

Mean auditory affective valence ratings of each patient group relative to the healthy older control group are plotted in Fig. 2. Across the sound stimulus set, mean valence ratings for each patient group showed a significant (*P* < .0001) positive correlation with control ratings. The AD group rated sounds overall as significantly (*P* < .05) less pleasant than the other groups; there were no other group differences for overall valence profile, although particular sounds were rated as significantly less pleasant by each of the patient groups relative to the healthy older control group (Fig. 2). Overall individual variation in affective valence ratings was significantly (*P* < .0001) increased in each of the patient groups relative to the healthy older control group (Supplementary Table 1 and Supplementary Fig. 1).

### 3.2 Pupillometric data

**Fig. 3:**
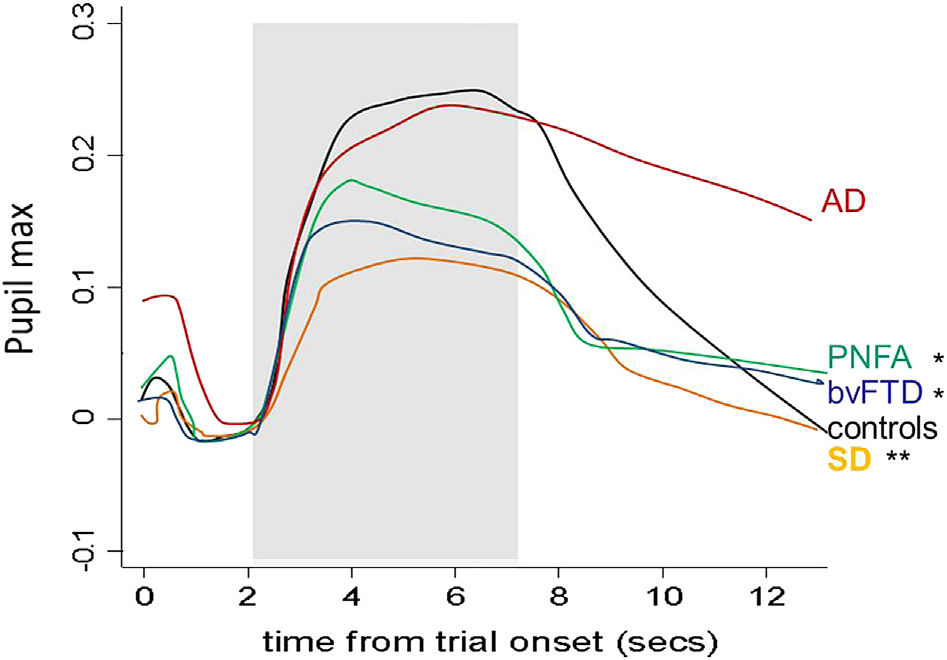
The mean time course of pupil response, pupil_max_ (the log ratio of maximal pupil area to baseline pupil area) over all trials is plotted for each participant group; sound stimulus presentation is indicated by the gray rectangle. Mean pupil responses were normal in the AD group, significantly reduced in the bvFTD and PNFA groups relative to the control and AD groups (^∗^*P* < .05) and significantly reduced in the SD group relative to all other groups (^∗∗^*P* < .05). AD, Alzheimer's disease; control, healthy older control group; bvFTD, behavioral variant frontotemporal dementia; PNFA, progressive nonfluent aphasia; SD, semantic dementia.

For all participant groups, pupil dilatation began around 0.25 s after sound onset and peaked around 1.5 to 2.0 s (Fig. 3). Baseline pupil size did not differ significantly between groups; the bvFTD, SD, and AD groups showed a reduction of baseline pupil size but not pupil_max_ over the course of the experiment. Mean pupil_max_ values over the entire sound stimulus set (indexing overall pupil reactivity to sound) were normal in the AD group but significantly (*P* < .001) reduced relative to both healthy controls and the AD group in the other patient groups; the SD group showed a smaller mean overall pupil_max_ response than all other groups and correspondingly smaller overall individual variability in pupil responses (all *P* < .05).

**Fig. 4:**
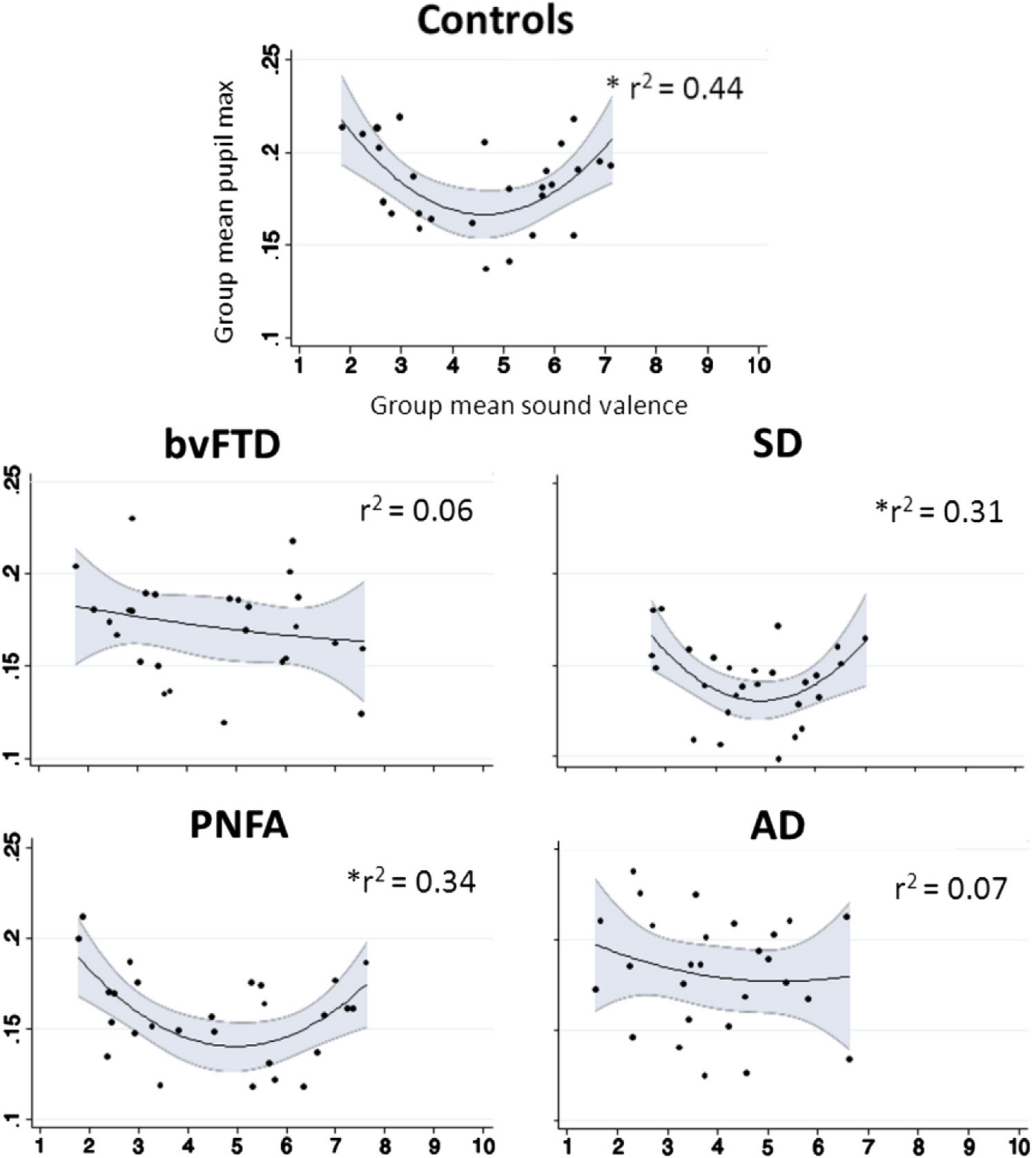
Group mean pupil_max_ (log ratio of maximal pupil area to baseline pupil area) in response to each stimulus sound plotted against own group mean affective valence (pleasantness) ratings, for each participant group. Valence ratings are on a Likert scale where 1 and 10 indicate most unpleasant and most pleasant, respectively. Quadratic regression lines of best fit with 95% confidence intervals (shaded gray zones) and corresponding r^2^ values are shown. ^∗^Significant (*P* < .05) correlations between pupil response and sound valence; AD, Alzheimer's disease; bvFTD, behavioral variant frontotemporal dementia; control, healthy older control group; PNFA, progressive nonfluent aphasia; SD, semantic dementia.

The healthy older control group showed a significant curvilinear relation (r^2^ = 0.44, *P* = .01) between pupil_max_ and affective valence ratings, with significantly greater pupil responses to both highly pleasant and unpleasant sounds than to neutral sounds (Fig. 4). When referenced to the affective valence ratings for the corresponding patient group, both the PNFA group (r^2^ = 0.34, *P* < .01) and the SD group (r^2^ = 0.31, *P* = .02) but not the other patient groups showed significantly increased pupil responses to highly valenced sounds (Fig. 4). This correlation was lost in the SD group if pupil responses were referenced to healthy control (rather than patients' own) valence ratings. Coefficients of the relation between pupil_max_ and affective valence did not differ significantly between groups. There was wide individual variability of pupil responses across the sound stimulus set in all participant groups (Supplementary Fig. 2); the magnitude of this variation in pupil response did not differ significantly between groups.

Pupillometric and behavioral valence rating profiles of syndromic groups relative to healthy older controls are summarized in Table 2.

### 3.3 Associations with general disease measures and auditory semantic function

There was no evidence that affective valence ratings, overall pupil reactivity, or pupil responses to sound valence correlated with disease severity (as indexed by nonverbal executive impairment, MMSE score or symptom duration), peripheral hearing function, or medication use.

The healthy older control group achieved subceiling scores on the sound pair semantic classification task; relative to controls, the PNFA and AD groups showed no auditory semantic deficit whereas both the SD and bvFTD groups showed significantly (*P* < .01) impaired performance, and the SD group performed significantly worse than the PNFA group (*P* = .05) (Table 1). Auditory semantic classification scores were significantly correlated with mean sound pair affective valence in the healthy older control group (*P* < .005) and in the bvFTD group (*P* < .05), such that more highly valenced sound pairs were classified more accurately; this correlation did not differ significantly between the healthy control and bvFTD groups, and was not present in other syndromic groups.

## 4 Discussion

Here we have shown that, relative to healthy older individuals, patients with canonical dementia syndromes have distinctive and partly dissociable profiles of autonomic (pupillary), behavioral affective, and cognitive responses to emotionally salient nonverbal sounds (Table 2). Patients with typical AD showed retained overall autonomic (pupillary) reactivity to sound but abnormal behavioral coding of auditory emotional salience, tending to rate sounds as generally more unpleasant than other syndromic groups. In contrast, patients with FTLD syndromes collectively mirrored this pattern, showing retained behavioral coding of emotional salience but impaired overall autonomic reactivity to sound. A more complex picture was evident in the relations between autonomic and behavioral emotional salience responses across syndromes: both AD and bvFTD showed loss of the normal coupling of autonomic and behavioral salience coding, whereas this was retained in PNFA. Although SD was associated with retained coupling of autonomic with behavioral responses as indexed by patients' own valence ratings, this coupling was lost if referenced to healthy control ratings, suggesting a distortion of the cognitive valuation of sounds. These performance profiles are in line with the auditory semantic deficits exhibited by both the bvFTD and SD groups.

Our findings corroborate and help to refine previous evidence concerning autonomic and affective reactivity to sounds and other emotional stimuli in neurodegenerative syndromes [13], [31], [32], [34], [35]. The syndromic profiles identified here together suggest a fractionated organization of auditory salience processing. Neuroanatomical correlates have not been defined in the present study, however, various candidate brain substrates have been identified in previous work, comprising distributed cortico-subcortical brain networks that are blighted in these neurodegenerative diseases [8], [23], [47], [48], [49]. The reciprocal interaction of “antagonistic” large-scale brain networks (in particular, the salience network implicated in the pathogenesis of bvFTD and the so-called “default mode network” implicated in AD), graded activity within these networks, and involvement of integrative “hubs” including insula, cingulate, and amygdala [8], [23] would allow for both overlap and divergence of pathophysiological profiles of auditory salience processing among dementia syndromes, as observed here (Table 2). The marked impairment of overall autonomic reactivity in SD here is consistent particularly with the severe involvement of central autonomic network hubs in amygdala and insula in this syndrome [23], [28]. The broadly similar profile in bvFTD is predicted from its closely overlapping anatomical signature, modulated by greater involvement of fronto-insular salience circuitry [8]. The present data suggest that bvFTD and AD have complementary disconnections of affective evaluation from autonomic integrative and effector processes, also in line with previous predictions [8], [23]. The PNFA syndrome is more anatomically and pathologically heterogeneous, with the predominant involvement of more dorsal and lateralized peri-Sylvian networks [21] perhaps accounting for its milder phenotype here.

These pupillometric and behavioral data broadly support the hypothesis that profiles of auditory emotional salience processing are altered in canonical dementia syndromes. The evidence for syndrome stratification was more qualified. On clinical grounds, bvFTD and SD were predicted to have the most marked derangements of emotional salience processing, yet patients with AD here showed abnormal affective coding of sounds: this may constitute a marker of heightened behavioral sensitivity to emotional stimuli underpinned by relative enhancement of salience network activity, recently proposed as a hallmark of AD [8], [14]. We present these findings with certain caveats. Individual variation in pupil responses and affective valence ratings was substantial and heightened in the patient cohort compared with healthy older individuals. Moreover, although affective rating profiles of the bvFTD, SD, and PNFA groups were similar overall to the healthy control group, particular sounds elicited discrepant valence ratings in these patient groups (Fig. 2 and Supplementary Table 1): it remains unclear whether this is simply a sampling issue or whether these sounds might tap more subtle disease-associated alterations in emotional salience coding.

This study has several limitations that suggest directions for future work. Group sizes were relatively small; the validity of the autonomic and behavioral metrics we have identified should be assessed in larger cohorts incorporating defined molecular pathologies and longitudinally, to define the time course of physiological alterations over the evolution of these diseases, including presymptomatic carriers of pathogenic mutations. The neuroanatomical correlates of the autonomic and behavioral metrics identified here remain to be defined: functional neuroimaging paradigms, ideally incorporating dynamic techniques such as magnetoencephalography with autonomic correlation will enable further evaluation of candidate brain mechanisms (Fig. 4). Ultimately, pathological correlation including detailed histomorphometry of key components of central autonomic circuitry will be required to establish the sensitivity and specificity of physiological markers for particular tissue pathologies and to define their brain substrates directly. Emotional sounds and pupillometry measures should be assessed alongside alternative stimulus paradigms and autonomic effector modalities tailored for particular behavioral signatures and diseases, and specific components of the affective response (in particular, valence and arousal) should be differentiated [8], [23], [50]. Autonomic indices will need to be correlated with clinical symptoms and disability to assess their functional relevance. Potential modulating effects of autonomically active drug classes should also be assessed to interpret clinical data in patients receiving these agents, and further, to test specific pathophysiological hypotheses (concerning, for example, aberrant reward processing [7]), and to dissect the relative contributions of sympathetic and parasympathetic control mechanisms.

Acknowledging these caveats, the present findings suggest that emotional sounds are a promising and versatile model for the analysis of salient environmental signals in neurodegenerative disease. The behavioral changes associated with aberrant reward processing and social disintegration are inherently difficult to define and quantify using conventional psychometric techniques, yet core to FTLD syndromes and increasingly recognized in a range of other neurodegenerative diseases including AD [7], [8], [14], [16]. Such behavioral alterations may reflect the breakdown of pathophysiological mechanisms that normally integrate sensory salience coding and cognitive evaluation. Our findings suggest that physiological phenotyping using salient sensory signals such as sounds may help to define these abnormal mechanisms, with implications for future diagnostic biomarker development and treatment strategies.

**Research in context**

1. Systematic review: We performed a PubMed search for relevant articles published in English using the terms “autonomic”, “pupillometric”, and “auditory” in conjunction with relevant dementia syndrome identifiers. Identified articles are cited accordingly.
2. Interpretation: Our findings support nonverbal sound as a useful model of sensory salience processing that integrates autonomic (physiological) with behavioral and cognitive mechanisms in canonical dementia diseases. The findings help reconcile a number of previous observations in these diseases.
3. Future directions: “Physiological phenotyping” of dementias provides a framework for testable hypotheses that should direct future studies. Examples include longitudinal analyses incorporating presymptomatic mutation carriers, to test the hypothesis that the physiological alterations of salience coding are sensitive markers of disease; and correlation with multimodal neuroimaging, to test the hypothesis that physiological markers reflect the specific disintegration of distributed neural networks.

## Figures and Tables

**Table 1:** Demographic, clinical, and neuropsychological characteristics of participant groups

Characteristic	Healthy controls	bvFTD	SD	PNFA	AD
General					
No. in group∗	26	14	10	12	10
Handedness (right:left)	25:1	13:1	8:2	11:1	10:0
Gender distribution (male:female)	12:14	11:3†	6:4	3:9	5:5
Age (yrs): mean (range)	67 (57–74)	66 (52–84)	65 (56–78)	68 (57–79)	66 (60–78)
Education score	17 (2)	15 (3)	15 (3)	15 (3)	15 (2)
Symptom duration (yrs)	NA	8.8 (6)†^,^‡^,^§	5.2 (2)	4.8 (2)	5.3 (2)
No. receiving AchEI/antidepressants	NA	6/4	1/3	2/3	9/2
MMSE (range)	30 (29–30)	25 (18–30)	**21 (9–29)**	28 (27–29)	**25 (21–29)**
IQ					
Verbal	123 (8)	**89 (20)**	**80 (18)**§	**77 (15)**§	**101 (14)**
Performance	119 (14)	**97 (17)**	110 (17)	**98 (17)**	**89 (16)**
Episodic memory					
RMT words (/50)	47 (3)	**35 (6)**	**32 (7)**	40 (8)	**30 (5)**||^,^†
RMT faces (/50)	44 (4)	**34 (6)**	**38 (8)**	**38 (5)**	**32 (5)**
Executive function					
Stroop word	21 (4)	**27 (9)**	27 (9)	**50 (14)**||^,^‡^,^§	**31 (9)**
Stroop inhibition	57 (16)	**94 (42)**	77 (32)	**118 (51)**	**116 (47)**
Digit span reverse (max)	5 (1)	5 (1)	6 (2)	**3 (1)**‡	5 (2)
Spatial span reverse (max)	7.6 (2)	**5.6 (2)**	**5.6 (2)**	**4.7 (1)**	**7.9 (2)**
Visuoperceptual function					
VOSP (/20)	18 (2)	17 (2)	16 (3)	16 (2)	**16 (2)**
Semantic processing					
BPVS (/150)	148 (2)	**132 (15)**	**99 (45)**||^,^§	**132 (24)**	**140 (8)**
Sound classification task¶ (45)	40 (5.2)	**35 (10.9)**	**35 (8.1)**†	38 (6.2)	38 (7.1)

Abbreviations: bvFTD, behavioral variant frontotemporal dementia; SD, semantic dementia; PNFA, progressive nonfluent aphasia; AD, Alzheimer's disease; AchEI, treatment with an acetylcholinesterase inhibitor; MMSE, Mini-Mental State Health Examination; IQ, intelligence quotient; NA, not applicable; RMT, Recognition Memory Test; VOSP, Visual Object and Space Perception battery; BPVS, British Picture Vocabulary Scale.

NOTE. Maximum total scores are shown (where applicable) after relevant neuropsychological tests; mean (standard deviation) data are shown unless otherwise indicated. Significant group deficits (*P* < .05) versus the healthy older control group are shown in bold. Other significant differences (*P* < .05) between groups are indicated by superscripts symbols and the explanation for these are provided below.

∗ General neuropsychological data not available for two patients in the PNFA group and one patient in the AD group.

† PNFA.

‡ SD.

§ AD.

|| bvFTD.

¶ Experimental nonverbal auditory semantic test (see text).

**Table 2:** Summary of syndromic profiles of emotional sound processing in patients relative to healthy controls

Disease group	Pupil responses		Valence rating	Semantic performance†
Overall reactivity	Valence coupling∗
bvFTD	Impaired‡	Impaired	Preserved	Impaired
SD	Impaired§	Preserved||	Preserved	Impaired¶
PNFA	Impaired‡	Preserved	Preserved	Preserved
AD	Preserved	Impaired	Impaired§	Preserved

Abbreviations: bvFTD, behavioral variant frontotemporal dementia; SD, semantic dementia; PNFA, progressive nonfluent aphasia; AD, Alzheimer's disease. See text for details.

∗ Correlation of pupil response with affective sound valence ratings by that group.

† Nonverbal auditory semantic classification task.

‡ Also relative to AD group.

§ Relative to all other groups.

|| Impaired if referenced to healthy control (rather than patients' own) affective ratings.

¶ Also relative to PNFA group.
