# Supplementary material for: Physiological phenotyping of dementias using emotional sounds
Source: Alzheimers Dement (Amst). 2015 Apr 22;1(2):170–8. doi: 10.1016/j.dadm.2015.02.003 (PMC4629103; doi:10.1016/j.dadm.2015.02.003)
Supplement: Online Supplementary Material [file mmc1.docx]

**SUPPLEMENTARY MATERIAL**

**Physiological phenotyping of dementias using emotional sounds,** by PD Fletcher et al.

**Selection and pre-processing of sound stimuli**

In an initial pilot study, 20 healthy younger individuals (median age 28 years (range 23-37), six male) were asked to identify and rate affective valences for a set of 180 nonverbal sounds. Sounds were derived from publically-available sound libraries (www.freesound.org and www.freesfx.co.uk) to sample a wide range of sources (human, animal, environmental, mechanical) and emotional valences. Ratings were on a Likert scale (1, very unpleasant; 10, very pleasant). Sounds that were misidentified by two or more (>10%) of the healthy young pilot control group were excluded, yielding a subset of sounds that were intrinsically highly familiar and identifiable; a mean affective valence rating derived from the pilot control group was assigned to each of the sounds meeting the identifiability criterion and used in constructing the experimental battery. Affective valence category ranges for sounds in the final stimulus set were as follows: ‘unpleasant’, range 1.6-2.9; ‘neutral’, range 3.3-5.7; ‘pleasant’, range 6.2-7.6. Following completion of experimental testing, the healthy older control participants rated each of the sounds for identifiability; the pilot healthy younger group and the healthy older control group had strongly correlated sound identifiability ratings, which were all identified correctly by >90% of healthy older controls. Sound valence categories had similar overall identifiability ratings, and there was no correlation between valence and identifiability ratings over the sound stimulus set. Stimulus characteristics are summarised in Table S1.

All sound samples were converted to digital wavefiles, sampled at 44.1 kHz; each sound was edited to five seconds duration (brief, periodic sounds such as hiccoughs were repeated within this interval) with fixed mean overall intensity (rms value).

Loudness changes provoke pupil dilatation in healthy individuals as part of a response to environmental salience ([Steiner and Barry, 2011](#_ENREF_54)) and might therefore potentially confound interpretation of auditory affective valence effects: we therefore measured the peak volume of each sound as played through the experimental sound delivery system using a sound level meter for incorporation as a nuisance covariate in subsequent analyses.

**Pre-processing of pupillometric and behavioural data**

In off-line analysis, pupil responses were calculated and artefacts were identified and removed using a customised algorithm in Stata12^®^. This algorithm calculated maximal pupil response as change from baseline pupil area for each trial; baseline values were calculated as the mean value over the initial two second silent interval of the trial. Artefacts were chiefly blinks, easily detected due to their characteristic temporal trajectory; pupil data were discarded for the interval 50 msec prior to 750 msec following an artefact, to allow for completion of an ensuing light reflex (as determined from data collected in the healthy young control pilot group). In a simple regression model, the total proportions of data points removed due to artefacts did not differ significantly (p>0.05) between sounds or between experimental groups. Maximum pupil area during a given trial was positively correlated with pupil area during the brief silent interval. In order to avoid this potentially confounding influence, the log ratio of maximal pupil area to baseline pupil area was used as the metric of pupil response for each trial (pupil**_max_**). Participant sound affective valence ratings as spatial positions along the pictorial on-screen Likert scale were converted to numerical values between 1 (very unpleasant) and 10 (very pleasant) for further analysis.

| **Trial**  **no.** | **Sound name** | **Valence categ** | **Young controls** | **Older controls** | | | **bvFTD** | | **SD** | | **PNFA** | | **AD** | |
| --- | --- | --- | --- | --- | --- | --- | --- | --- | --- | --- | --- | --- | --- | --- |
|  |  |  | mean valence | ident | mean valence | s.d. | mean  valence | s.d. | mean valence | s.d | mean valence | s.d. | mean valence | s.d. |
| 1 | infant wailing | 1 | 2.6 | 100 | 2.9 | 1.5 | 4.6 | 3.1 | 4.1 | 2.5 | 3.1 | 2.2 | **5.0↑** | 2 |
| 2 | paper rustling | 2 | 4.7 | 90 | 4.4 | 1.4 | 4.2 | 2 | 3.4 | 2.4 | 4.3 | 2.4 | 4 | 1.7 |
| 3 | baby cooing | 3 | 7.8 | 100 | 6.7 | 1.4 | 5.4 | 2.7 | 6.6 | 2.5 | 5.6 | 2.9 | 6.6 | 1.7 |
| 4 | water trickling | 3 | 6.6 | 100 | 6.1 | 1.5 | 6.3 | 2.2 | 6.4 | 1.8 | 5.2 | 2.3 | 5.4 | 1.8 |
| 5 | bees humming | 1 | 1.9 | 90 | 2.8 | 1.2 | 3.4 | 2.2 | 4.4 | 2.8 | 2.3 | 0.9 | 3.3 | 0.8 |
| 6 | woman yawning | 2 | 4.9 | 100 | 5.6 | 0.9 | 6.1 | 2.9 | 6.5 | 2.3 | 5.1 | 2.6 | 5 | 1.9 |
| 7 | person spitting | 1 | 1.7 | 90 | 2.5 | 1.4 | 3.2 | 1.4 | 3.5 | 2.8 | 2.4 | 1.3 | 2.3 | 1.1 |
| 8 | child hiccoughing | 2 | 4.2 | 90 | 3.3 | 1.1 | 3.4 | 1.9 | 4.3 | 3.1 | 3.4 | 1.9 | 3.4 | 1.5 |
| 9 | fizzy drink poured | 3 | 7.6 | 100 | 6.4 | 1.3 | 5.9 | 2.3 | 5.8 | 1.9 | 6.1 | 2.6 | 5.4 | 2.1 |
| 10 | thunder | 2 | 5.2 | 100 | 5.1 | 2.2 | **2.8↓** | 2.0 | 4 | 2.5 | 4.5 | 2.4 | **3.6↓** | 1.6 |
| 11 | waves lapping | 3 | 8.4 | 100 | 5.8 | 1.9 | 6 | 2.8 | 4.8 | 2.4 | 6.7 | 1.9 | **3.5↓** | 1.9 |
| 12 | telephone receiver replace | 1 | 1.8 | 90 | 3.6 | 1.2 | **2.5↓** | 1.1 | 4.1 | 1.6 | 3.4 | 2.4 | 3.7 | 1.7 |
| 13 | man belching | 1 | 1.6 | 90 | 2.6 | 1 | 2.4 | 1.6 | 3.8 | 2.6 | **1.9↓** | 0.7 | 2.2 | 1.2 |
| 14 | woman giggling | 3 | 7.4 | 100 | 6 | 1.7 | 7 | 2.2 | 6 | 2.5 | 6.9 | 1.9 | 4.8 | 1.8 |
| 15 | train horn | 2 | 4.7 | 90 | 5.8 | 1.3 | 5.2 | 2.7 | 5.6 | 2.7 | 5.5 | 2.7 | **3.7↓** | 1.6 |
| 16 | woman humming | 3 | 7 | 100 | 6.9 | 1.2 | 7.5 | 1.6 | 5.7 | 3.8 | 7.1 | 1.4 | 6.6 | 2.7 |
| 17 | car horn | 1 | 1.9 | 100 | 3.4 | 1.1 | 3.5 | 1.9 | 4.3 | 2.7 | 3 | 2 | **2.3↓** | 0.6 |
| 18 | woman clearing throat | 2 | 4.6 | 100 | 4.7 | 0.7 | 4.8 | 2.1 | 5.1 | 2.3 | **3.1↓** | 1.6 | 4.5 | 1.6 |
| 19 | man vomiting | 1 | 1.4 | 100 | 1.8 | 0.8 | 1.8 | 0.5 | 2.7 | 2.3 | 1.8 | 0.6 | 1.7 | 0.7 |
| 20 | telephone ringing | 2 | 4.8 | 90 | 4.4 | 0.9 | **3.1↓** | 1.6 | 5.7 | 2.5 | 5.5 | 1.9 | 3.8 | 1.2 |
| 21 | stream burbling | 3 | 7.7 | 100 | 6.4 | 1.6 | 6.1 | 2.7 | 6.1 | 2.2 | 6.5 | 2.4 | **4.6↓** | 1.1 |
| 22 | infant sneezing | 2 | 4.5 | 90 | 3.2 | 1.3 | 2.9 | 1.3 | 2.9 | 1.3 | 2.9 | 1.1 | 2.7 | 1 |
| 23 | woman crying | 1 | 1.8 | 100 | 3 | 0.9 | 2.9 | 1.4 | 2.8 | 1.3 | 2.4 | 1.4 | 2.5 | 1.5 |
| 24 | waves crashing | 3 | 7.6 | 100 | 5.8 | 1.6 | 5.2 | 3.0 | **3.6** | 1.6 | 5.4 | 2.5 | 4.3 | 2.8 |
| 25 | horse trotting | 3 | 6.8 | 100 | 6.4 | 1.2 | 6.2 | 1.8 | 5.3 | 3.1 | 7.2 | 1.6 | 5.8 | 1.9 |
| 26 | person brushing teeth | 2 | 5 | 100 | 4.6 | 0.9 | 4.9 | 2.3 | 4.8 | 2 | 5.3 | 2.6 | 5.1 | 1.5 |
| 27 | mosquito | 1 | 1.4 | 100 | 2.6 | 1.2 | 3.7 | 2.2 | 4.5 | 3.1 | 2.5 | 1.9 | 3.2 | 1.5 |
| 28 | woman screaming | 1 | 1.3 | 100 | 2.3 | 0.9 | 2.1 | 0.8 | 2.8 | 1.5 | 2.4 | 2 | 1.6 | 0.6 |
| 29 | car engine idling | 2 | 4.8 | 100 | 5.1 | 1.2 | 5 | 2.3 | 5.3 | 2.2 | 4.5 | 2.6 | 4.2 | 1.8 |
| 30 | baby laughing | 3 | 8.8 | 100 | 7.1 | 1.1 | 7.6 | 1.8 | 7 | 2.3 | 7.6 | 1.7 | 6.6 | 2.2 |

**Table S1.** Experimental playlist and sound stimulus psychological characteristics for the pupillometry experiment. Sound stimuli are listed in trial presentation order. The healthy younger control group (n=20, median age 28 years (range 23-37), 6 male) participated in an initial pilot experiment on a larger set of 180 nonverbal sounds that were rated for affective valence (pleasantness, 1 - 10) on a Likert scale. For participant groups in the main experiment, affective valence ratings (mean and standard deviation, s.d.) assigned to each individual sound by the relevant group are shown. **categ**, valence category: 1= negative, 2= neutral, 3= positive; **ident**, for each of the 30 sounds, the proportion of the healthy older control group correctly identifying that sound is shown. Mean proportion of sounds correctly identified were similar between valence categories (valence category 1, 96%; category 2, 96% ; category 3, 100%). Patient group valence values significantly different from the older control group are coded in bold; arrows indicate whether the mean value is larger (↑) or smaller (↓) than that of the older control group. The first three practice trials are shaded in grey. **AD**, Alzheimer’s disease; **bvFTD**, behavioural variant frontotemporal dementia; **PNFA**, progressive nonfluent aphasia; **SD**, semantic dementia.

| ***‘same’ sound pairs*** | | valence 1 | valence 2 | mean valence |
| --- | --- | --- | --- | --- |
| woman crying | woman screaming | 1.8 | 1.3 | 1.6 |
| man shouting in pain | man vomiting | 2.1 | 1.4 | 1.7 |
| car crash sounds | car horn | 1.9 | 1.9 | 1.9 |
| car horn | car skidding | 1.9 | 2.2 | 2.1 |
| man sobbing | person breaking wind | 2.3 | 2.5 | 2.4 |
| infant wailing | infant sobbing | 2.6 | 2.3 | 2.4 |
| person coughing | person snoring | 3.6 | 2.8 | 3.2 |
| woman clearing throat | woman yawning | 4.6 | 4.9 | 4.7 |
| geese honking | goose wings flapping | 5.1 | 4.2 | 4.6 |
| grandfather clock chime | grandfather clock ticking | 6.5 | 5.6 | 4.6 |
| train travelling on tracks | train horn | 5.5 | 4.7 | 5.1 |
| cockerel crowing | hen clucking | 5.9 | 5.7 | 5.8 |
| pigeon wings flapping | pigeon cooing | 5.7 | 6.3 | 6.0 |
| woman giggling | woman humming | 7.4 | 7.0 | 7.2 |
| stream burbling | waves lapping | 7.7 | 8.4 | 8.1 |
| baby cooing | baby laughing | 7.8 | 8.8 | 8.3 |
| *Condition mean valence* |  |  |  | *4.4* |
| ***‘different’ sound pairs*** | | valence 1 | valence 2 | mean valence |
|  |  |  |  |  |
| mosquito | woman screaming | 1.4 | 1.3 | 1.3 |
| man vomiting | clock alarm bell | 1.4 | 2.2 | 1.8 |
| woman crying | car crash sounds | 1.8 | 1.9 | 1.8 |
| man sobbing | bees humming | 1.8 | 1.9 | 1.9 |
| telephone receiver replace | woman coughing | 1.8 | 2.1 | 1.9 |
| infant wailing | man wheezing | 2.6 | 2.1 | 2.3 |
| man shouting in pain | car horn | 2.1 | 2.9 | 2.5 |
| car skidding | man snoring | 2.2 | 2.8 | 2.5 |
| car alarm disarmed | shovel on metal | 3.3 | 4.2 | 3.7 |
| telephone dial tone | man clearing throat | 3.4 | 4.1 | 3.7 |
| cat whining | puppy yelping | 4.1 | 3.6 | 3.8 |
| paper tearing | child hiccoughing | 3.9 | 4.2 | 4.0 |
| telephone ringing | person breathing | 4.8 | 4.2 | 4.5 |
| paper rustling | woman clearing throat | 4.7 | 4.6 | 4.6 |
| infant sneezing | engine running | 4.5 | 4.8 | 4.6 |
| person brushing teeth | train horn | 5.0 | 4.7 | 4.8 |
| shovel digging gravel | car window winder | 5.3 | 4.7 | 5.0 |
| person clapping hands | dog barking | 5.3 | 5.1 | 5.2 |
| horse whinnying | woman yawning | 5.5 | 4.9 | 5.2 |
| child yawning | geese honking | 5.7 | 5.1 | 5.4 |
| pigeon wings flapping | person chewing | 5.7 | 5.3 | 5.5 |
| hen clucking | man sighing | 5.7 | 5.3 | 5.5 |
| fizzy drink can opened | coin dropped on table | 6.2 | 6.2 | 6.2 |
| pigeon cooing | fingers clicking | 6.3 | 6.2 | 6.3 |
| horse trotting | person walking on gravel | 6.8 | 6.0 | 6.4 |
| woman humming | water trickling | 7.0 | 6.6 | 6.8 |
| waves crashing | woman giggling | 7.6 | 7.4 | 7.5 |
| baby cooing | stream burbling | 7.8 | 7.7 | 7.8 |
| baby laughing | waves lapping | 8.8 | 8.4 | 8.6 |
| *Condition mean valence* |  |  |  | *4.5* |

**Table S2.** Experimental stimuli for the auditory semantic classification experiment: pairs in ‘same’ and ‘different’ conditions, here ordered by mean affective valence assigned each sound pair; sounds in each pair were closely matched for valence (within 1 rating point). Valence ratings are derived from the pilot healthy younger control group (see legend Table S1). Sound pairs were presented in randomised condition order during the experiment.

**Figure S1.** Variation in individual affective valence ratings (relative to group mean rating) for each stimulus sound plotted against healthy older control mean valence ratings, for each participant group. Ratings are on a Likert scale where 1 and 10 indicate most unpleasant and most pleasant, respectively; quadratic regression lines of best fit are shown. Compared with the healthy older control group, the SD group showed significantly increased variability of valence ratings over the whole sound stimulus set, whereas the AD group showed significantly increased variability rating positively valenced sounds and the bvFTD and PNFA groups showed increased variability rating neutral sounds (all p<0.01). AD, Alzheimer’s disease; bvFTD, behavioural variant frontotemporal dementia; control, healthy older control group; PNFA, progressive nonfluent aphasia; SD, semantic dementia.

**Figure S2.** Individual pupil**_max_** (log ratio of maximal pupil area to baseline pupil area) in response to each stimulus sound plotted against group mean affective valence ratings, for each participant group. Ratings are on a Likert scale where 1 and 10 indicate most unpleasant and most pleasant, respectively; quadratic regression lines of best fit with 95% confidence intervals (shaded grey zones) are shown. AD, Alzheimer’s disease; bvFTD, behavioural variant frontotemporal dementia; Control, healthy older control group; PNFA, progressive nonfluent aphasia; SD, semantic dementia
